# Three Weeks in a Hospital

**Published:** 1892-01-16

**Authors:** 


### TURNING OVER NEW LEAVES.

### Three Weeks in a Hospital.

THIS is a book* so interesting and well written as to deserve a wide popularity and circulation. The story is graphically told, true to life, and so constructed that when commenced it will not easily be put down until finished. There is much dry humour to be found in its pages, and it would be well for the public and the hospitals if it could find its way into every household. The facts are reliable, and those who know most about hospitals will welcome its publication, because the story brings out clearly the actual routine and system to be found in the present day in every English hospital supported by voluntary contributions which has any title to the name. A knowledge of the world convinces the most sceptical that critics, as a rule, are mainly drawn from the least useful and intelligent members of society. If a person feels his own ignorance and incapacity, he or she not infrequently discovers that the easiest method to secure notice for themselves is to criticise harshly the action and work of their fellows, who they are conscious are superior to, and more worthy than, themselves. This is an age when sensation has assumed a popularity harmful to the best interests of the race. A certain class of contributors to a few journals have discovered this fact, and trade upon it by writing spicey but carping paragraphs about persons and institutions of whom they in reality know little or nothing. Hence a true account of the inner life of the great hospitals such as this book contains must do good. It will, we hope, attract wide editorial notice and so lead to a wiser and wider interest in hospital work, to be followed at no distant date by the general adoption of a practice whereby the work and results of our hospitals will be personally tested by members of the press, who will thus become frequent visitors at these institutions. Then, and then only, can they realise for themselves what a high standard of efficiency prevails within their walls in the present day. The story is based upon the experience of a certain Baronet, who is badly injured, in the London streets, and is carried into a large ward of one of the general hospitals. It is very interesting and quite accords with our own experience to find that the Baronet regrets his removal to a private ward when a vacancy is there made for him because he misses the life and companionship of his humble but well conducted fellow patients whom he leaves behind him. It is further worthy of notice that the Baronet asks for the screens to be moved away from his bed, because he prefers to see and enter into the life of the great ward in which he finds himself on regaining consciousness. Again, when he is dismissed from the hospital and returns to Carew Hall there is an amusing but true description of the discomfort resulting from the feeling that "I have scarcely closed my eyes all night; and not a soul has troubled to come near me. Why," glancing savagely at his watch, which his valet had forgotten to wind up, "it must be nearly seven o'clock, and not a sign of breakfast." The patient had grown accustomed to the early habits of the hospital, and positively resented a return to the luxurious laziness of his home life. It is well to bear this in mind, having regard to the complaints which we hear from time to time of the hardships engendered by the early hours enforced by hospital necessities and discipline. The book, then, is a good book, a real living picture of life in a hospital; one which must increase the popularity of these useful institutions and so render great public service. The typography, illustrations, and general get-up of the volume are excellent, and we can cordially commend it to our readers. Every hospital and ever hospital worker, patient, and governor, should possess a copy of "Hors de Combat."

* "Hors de Combat; or, Three Weeks in a Hospital," Founded on Facts. By Gertrude and Ethel Armitage Southam. Illustrated. (Cassell and Co. Limited, London, Paris, and Melbourne.)

